# A qualitative investigation into pregnancy experiences and maternal healthcare utilisation among adolescent mothers in Nigeria

**DOI:** 10.1186/s12978-023-01613-z

**Published:** 2023-05-20

**Authors:** Christiana A. Alex-Ojei, Clifford O. Odimegwu, Lorretta F. C. Ntoimo

**Affiliations:** 1 Demography and Population Studies Programme, Schools of Public Health and Social Sciences, University of the Witwatersrand, Room 136, Robert Sobukwe Block, East Campus, Johannesburg, South Africa; 2 Department of Demography and Social Statistics, Faculty of Social Sciences Building, Federal University Oye-Ekiti, Oye-Ekiti, Ekiti State Nigeria

**Keywords:** Maternal healthcare utilisation, Adolescents, Sociocultural factors, Social support, Nigeria, Africa

## Abstract

**Background:**

Adolescent maternal healthcare utilisation is low in Nigeria, and little is understood about the pregnancy experiences and drivers of maternal healthcare utilisation among of adolescent girls. This study investigated the pregnancy experiences and maternal healthcare utilisation among adolescent mothers across Nigeria.

**Methods:**

The study used the qualitative design. Urban and rural communities in Ondo, Imo and Katsina states were selected as research sites. Fifty-five in-depth interviews were conducted with adolescent girls who were currently pregnant or had given birth to a child recently, and nineteen in-depth interviews were conducted with older women who were either mothers or guardians of adolescent mothers. Additionally, key informant interviews were conducted with five female community leaders and six senior health workers. The interviews were transcribed, and resulting textual data were analysed via framework thematic analysis using a semantic and deductive approach, with the aid of NVivo software.

**Results:**

The findings showed that the majority of unmarried participants had unintended pregnancies and stigma against pregnant adolescents was common. Social and financial support from family members, maternal support and influence, as well as healthcare preferences shaped by cultural and religious norms were the major drivers of maternal healthcare use among adolescent mothers, and the choice of their healthcare providers.

**Conclusions:**

Interventions to support adolescent mothers and increase maternal healthcare utilisation among them must focus on ensuring the provision of social and financial support for adolescent mothers, and should be culturally sensitive.

**Supplementary Information:**

The online version contains supplementary material available at 10.1186/s12978-023-01613-z.

## Introduction

Adolescent pregnancy is a common occurrence in Nigeria, as nearly one-fifth of adolescent girls aged 15–19 were either currently pregnant or had given birth to at least one child in 2018, and the adolescent fertility rate is 106 births per 1000 girls in this age group [1]. Pregnancy is often a risk-filled endeavour for adolescent girls, given their physiological development is still on-going. Pregnant adolescents are susceptible to anaemia, pregnancy complications, obstructed labour due to an incompletely developed pelvis, and other pregnancy-related morbidity. They are also more likely to have low birth-weight and preterm babies, as well as stillbirths [2–9]. Additionally, about 40% of deaths among girls aged 15–19 in Nigeria are from maternal causes [1]. In order to reduce the prevalence of adolescent pregnancies in the country, the 2007 Nigerian Adolescent Health Policy aimed to reduce the incidence of unwanted pregnancies by adolescent females by 50% by year 2015; this target, however was not achieved. In a more recent attempt to reduce adolescent pregnancy incidence, the Federal Ministry of Health in Nigeria in partnership with the World Health Organization inaugurated the Gender Adolescent School Health and Elderly Care (GASHE) programme to provide comprehensive sex education to adolescents between ages 13 and 18 in secondary schools across Nigeria. Adolescent pregnancies are more likely to be unintended in sub-Saharan Africa, especially when the mothers are unmarried [10, 11]. Adolescent girls often have poor sexual and reproductive knowledge, leading to unintended and unwanted pregnancies [12].

The risks notwithstanding, adolescent mothers have the lowest utilisation rates of maternal health services in Nigeria [13–17], as well as in other sub-Saharan African countries [18, 19]. Several factors influence maternal healthcare utilisation among adolescent mothers. Girls with higher socioeconomic status tend to have higher usage of maternal health services [20–24]. Adolescent mothers living in urban areas have higher maternal healthcare use than rural mothers [21, 25–27]. This is especially true when health facilities are distant in these rural areas [23, 28]. Furthermore, adolescent mothers who have high media exposure and reproductive health knowledge levels have higher maternal healthcare use [20–22, 24, 26, 27].

Literature shows that adolescent maternal healthcare utilisation is also influenced by sociocultural factors. For instance, unmarried adolescent mothers experience stigma regarding their pregnancies [11, 29–32], and the presence of this stigma may lead to suboptimal use of maternal healthcare services [11, 33]. Health worker attitudes also exert influence on maternal healthcare use among the youngest mothers, as previous studies show that harsh and unsympathetic health worker attitudes reduced maternal healthcare use among them [28, 31, 32, 34–37]. Studies in Ghana and Zimbabwe found that adolescent mothers are more able to use healthcare when they receive social support from their families during their pregnancy [34, 38]. Studies in Uganda and Bangladesh discovered that the adolescent mothers’ choice of healthcare provider was largely influenced by the type of healthcare preferred in the household and the wider communities where they lived, since they are more susceptible to influence from others due to their lack of experience [28, 39].

A few studies conducted on adolescent maternal healthcare utilisation in Nigeria have largely examined the socioeconomic and demographic determinants of maternal healthcare utilisation among this group of mothers using a quantitative approach [9, 26, 40, 41]. The previous studies which examined the experiences of adolescent mothers, as well as sociocultural factors such as stigma and pregnancy intention among them have been limited to single regions of the country [11, 42]. While findings from these studies have provided important information on the some of the factors that influence maternal healthcare use among adolescent mothers in the country, little is known about the features of the sociocultural environment peculiar to Nigeria that either help or hinder these mothers’ access to and use of MHC services.

Therefore, this study explores the pregnancy experiences of adolescent mothers across Nigeria, their maternal healthcare utilisation, and the sociocultural factors that either aid or hinder their utilisation of healthcare services. The study adopted an ethnographic qualitative approach to obtain richer information and more in-depth understanding of adolescent mothers’ lived experiences, and the reasons behind their maternal healthcare usage patterns. Furthermore, it compares the prevailing sociocultural factors in different regions that represent the three major ethnic groupings in the country, to examine similarities and/or differences in these sociocultural influences. This is important to policy and programme designers as Nigeria is a culturally and religiously diverse country, and interventions which work in one region may not do so in others. The study answers the following questions: (i) what are adolescent girls’ pregnancy experiences across Nigeria? (ii) what types of maternal healthcare do pregnant adolescents and new adolescent mothers make use of in Nigeria? and (iii) what are the factors that guide adolescent mothers’ maternal healthcare utilisation in Nigeria?

### Theoretical framework

The study used the Andersen’s Behavioural Model of Healthcare Utilisation, which examines the factors that influence healthcare use in a population. The model has been previously used to study healthcare utilisation and various health outcomes, for example, mental-health seeking behaviour [43, 44], the healthcare cost of intimate partner violence [45], the influence of culture on health-seeking behaviour of older immigrants [46], patient-physician interaction [47], access to preventive healthcare [48], general health services utilisation [49] and maternal healthcare utilisation [26, 50].

The 2008 revision of the model was used as the theoretical framework for this study. While previous versions of the theory put forward predisposing, enabling and need factors as determinants of healthcare utilisation, this revision categorised these factors (predisposing, enabling and need) into individual and contextual levels. The study adapted the theory to examine the factors influencing maternal healthcare utilisation among adolescent girls in Nigeria. Using the Andersen framework, the study examined the predisposing, enabling and need factors that influenced maternal healthcare utilisation among adolescent girls at both the individual level and from their cultural environments.

## Materials and methods

The study was conducted as the qualitative component of an explanatory sequential mixed methods study, where quantitative data were first analysed, and qualitative findings were used to further explain and expand on the quantitative findings [51]. It used ethnographic qualitative methodology, using in-depth and key informant interviews to gather information from currently or ever pregnant adolescent girls, their mothers and guardians, and community leaders. The study was carried out in Nigeria, which is divided into six geopolitical zones, namely, North West, North East, North Central, South West, South East and South South, and 36 states, including the Federal Capital Territory in Abuja. Nigeria is a multi-ethnic country, with more than 250 recognised ethnic groups. The three major ethnic groups by population size are the Yoruba, Igbo and Hausa groups, with the Yoruba predominantly in the South West and parts of the North Central zones, the Igbo in the South East and the Hausa spread across the North Central, North West and North East zones of the country.

The study was conducted in three geopolitical zones that represent the major ethnic groups in Nigeria, namely the Hausa and Fulani ethnic groups in the North West zone, the Igbo ethnic group in the South East zone, and the Yoruba ethnic group in the South West zone. In each of the three zones, the state with the highest recorded pregnancy incidence as at the time the study was conducted was selected. Katsina State was selected from the North West zone, Imo State in the South East, and Ondo State in the South West. Additionally, Katsina State had the highest adolescent pregnancy rate in the entire country based on the available evidence at the time the study was conducted [52]. In each state, two study locations, one rural and one urban, were chosen to compare the pregnancy experiences and maternal healthcare utilisation of adolescent girls in urban and rural areas. The urban research sites chosen were Akure, the capital of Ondo State, Owerri the capital of Imo State, and Katsina Township, which is the capital of Katsina State. The rural sites selected were Aponmu in Ondo State, Assa in Imo State, and Majigiri in Katsina State. Ethical approval for the study was obtained from the University of the Witwatersrand Human Research Ethics Committee (non-medical), with protocol number H18/06/03.

All of the interviews were conducted using semi-structured interview guides. A pilot study was conducted in October 2018 in Ekiti State, South West Nigeria, to examine the validity of the interview guide, and ambiguous questions were rephrased in the final guide used for the study. The data collection was conducted between November 2018 and January 2019. Purposive sampling was used to recruit willing eligible participants in the selected communities into the study. The inclusion criteria for the study were all girls aged 15–19 living in the selected communities, who were either currently pregnant or had been pregnant or given birth to at least one childas adolescents; mothers or guardians of girls who currently were or had been adolescent mothers; or female community leader. Informed consent, as well as consent to audio-record their conversations, was obtained from all participants. For adolescent girls younger than age 18, parental consent was obtained from their parents or guardians, and willingness to participate was obtained from the respondents themselves. Due to the nature of the study, participants were offered distress counselling with a licensed clinical psychologist. Only one potential respondent was unable to participate in the study, as she became too emotional to proceed with the interview. Where respondents declined to be audio-recorded, their responses were transcribed directly during the interviews. The primary language of the interviews guides was English, though some interviews were conducted in Yoruba and Hausa languages in the South West and North West zones for respondents who could not or did not want to be interviewed in English. In such cases, the interview questions were translated into the relevant languages, and responses were translated during the transcription process as closely as possible to retain the true meanings of responses. In the South East zone, all interviews were conducted in English as that was the preferred language for respondents in that zone. The number of completed interviews varied across the various study sites based on the availability of willing participants. In total, fifty-five adolescent mothers, nineteen mothers and guardians of adolescent mothers, five female community leaders and six senior health workers participated in the interviews.

### Sociodemographic characteristics of participants

Table 1 shows the sociodemographic characteristics of the study participants. The adolescent mothers in the study were between 15 and 22 years old. In the South West region, 56.2% of participants were urban residents; 37.5% of participants had secondary education. More than half of participants were single, living with their parents or guardians (56.2%). The majority of respondents (68.8%) were Yoruba. All participants were Christians. In the North West region, the participants were evenly split between urban and rural areas. The majority of participants had no formal education (70.8%). The majority of participants (95.8%) were married. The majority of participants were Hausa (87.5%) and 12.5% were Fulani. All participants were Muslims. In the South East region, 80% of participants lived in the rural area; 26.7% had secondary education, Also, 26.7% of participants in this region were married, and 40.0% were single and living with their parents or guardians. All of the participants were Igbos and Christians.

**Table 1:** Sociodemographic characteristics of respondents

Sociodemographic characteristics	Region of residence (%)		
	South West (N = 16)	North West (N = 24)	South East (N = 15)
Place of residence			
Urban	56.2	50.0	20.0
Rural	43.8	50.0	80.0
Educational level			
None	0.0	70.8	0.0
Primary	12.5	4.2	6.6
Basic	18.8	0.0	26.7
Incomplete secondary	31.2	25.0	40.0
Secondary	37.5	0.0	26.7
Marital status			
Single	56.2	0.0	40.0
Formerly cohabiting with partner	0.0	0.0	13.3
Cohabiting with partner	43.8	0.0	13.3
Married	0.0	95.8	26.7
Widowed/divorced	0.0	4.2	6.6
Ethnicity			
Yoruba	68.8	0.0	0.0
Igbo	18.8	0.0	100.0
Idoma	6.2	0.0	0.0
Ebira	6.2	0.0	0.0
Hausa	0.0	87.5	0.0
Fulani	0.0	12.5	0.0
Religion			
Christianity	100.0	0.0	100.0
Islam	0.0	100.0	0.0

### Data analysis

Data analysis was conducted using a deductive approach, as a list of likely themes had been prepared which were derived from the theoretical and empirical background of the study. These pre-determined themes were then used in the construction of questions in the interview guides used. It also used the semantic approach, where data analysis focused mainly on the information explicitly provided by participants, and not the meanings behind them. The framework option of the codebook thematic analysis method was data analysis with the aid of the NVivo qualitative analysis software (version 12) [53–56]. Data analysis started by reading through the various interview transcripts and selecting the most relevant verbatim quotes for coding. These codes were then arranged according to themes, which were then classified either as major or sub-themes, using the domain summary method [57]. The same process was conducted for all interviews, that is, in-depth interviews with the adolescent mothers and their mothers and guardians, as well as the key informant interviews with community leaders and health workers. Data from the adolescent mother interviews were analysed first, followed by the mothers, the female community leaders and the health workers. The different perspectives from the various interviews were brought together using themes that were common across them. In total, two major themes and six sub-themes were identified.

## Results

### Pregnancy experience of adolescent girls

#### Pregnancy intention

There were differences in pregnancy acceptance among adolescent girls, depending on their marital status and region of residence. In the South West and South East regions where, early marriage is less common, fewer adolescent mothers reported being married, and for most of them who were either married or cohabiting with their partners, the pregnancy was their point of entry into marriage or at least cohabitation. For adolescent mothers in the North West region where early marriage was the norm, marriage generally preceded pregnancy.

Unmarried young women reported being unprepared for their pregnancies, with one describing at a mistake. Her mother’s decision for her to leave home and go and live with the father of her child is a common response to an adolescent pregnancy, as it is felt that girls who get pregnant at young ages are trying to prove that they are now adults, and as such, should be forced into the adult world of caring for their own home.

*We were both in school; he was my senior, he used to come and visit me, but we never planned that I would get pregnant.* (AK 02, 16, single, SW, Yoruba, urban, Christian, incomplete secondary).

*No, this was a mistake…...we were dating before the mistake happened, and my mother said I should leave her house and go to the person responsible for my pregnancy, and I went to live with him.* (AP06, 19, cohabiting, SW, Yoruba, rural, Christian, basic)

Married adolescent mothers, however described their pregnancies as wanted, since the expectation for them was that the next logical step after marriage would be pregnancy.

*Yes, it* (the pregnancy) *was* (planned) *as I am in my husband’s house…we decided it was time to have a child.* (AS05, 17, married, SE, Igbo, rural, Christian, secondary)

*Whoever gets married must expect pregnancy, so I expected it.* (MJ03, 18, married, NW, Hausa, rural, Muslim, no education)

However, there were adolescent mothers who had contrary opinions about their pregnancies. Here we see an unmarried adolescent mother in the South West who got pregnant on purpose to prevent an unwanted marriage.

*I planned to get pregnant for my boyfriend because my parents were trying to force me to marry someone else…so I thought if I get pregnant, they would not be able to force me to marry the man of their choice.* (AP02, 22, single, SW, Igbo, rural, Christian, secondary)

This young mother, despite being married and living in a community where early marriage was common, said that she would have preferred to wait a little longer after marriage before getting pregnant. This shows that even where early and childbearing are common, individual choice may differ from the norm.

*I never expected to be pregnant when I got married and it was just sudden.* (KT06, 19, married, NW, Hausa, urban, Muslim, incomplete secondary)

#### Experiences of stigma

Some adolescent mothers reported experiencing stigma especially via verbal abuse to varying degrees. Most of the mothers who reported stigma were unmarried mothers who were not living with their partners, although some married and cohabiting mothers reported being verbally abused. There was the general feeling that pregnancy was not acceptable among young girls, as they should be in school.

*Somebody came to tell me that some people said I have nothing to eat…that I was impregnated and the person responsible didn’t take care of me. That I was sent to school but became pregnant. I have fought with someone in this area when she asked me why I was pregnant when I should be in school, so I fought with her over why she would say such a thing to me.* (AK04, 19, single, SW, Yoruba, urban, Christian, incomplete secondary)

This adolescent mother’s experience reinforces the cultural impression that it is a shameful thing for a young girl to get pregnant while still living with her parents, and one of the major reasons why pregnant girls are asked to go and live with their baby’s father.

*Yes, I experienced it…some of them were talking about the fact that I was pregnant in my father’s house…some came to say it to my face.* (AS08, 20, single, SE, Igbo, rural, Christian, incomplete secondary)

For this adolescent mother, she looked much younger than she is, and due to her extremely young look, she attracted verbal abuse from a total stranger who even advised her to get an abortion as they felt she was too young to be carrying a pregnancy.

*Someone met me on the road and told me to go and abort my pregnancy, that I was too young to be pregnant…he didn’t know I’m married.* (AS12, 19, married, SE, Igbo, rural, Christian, basic)

Stigma was found to be tied to the perception of adolescent pregnancy by older community members. The different responses given by older women indicate that adolescent pregnancy was frowned upon among unmarried girls, and only seen as acceptable where the young girl was married.

This community leader’s response suggests that she viewed adolescent pregnancy through the lens of deviant behaviour, as girls who got pregnant did so after ignoring warnings about their lifestyle.

*No, the people in this area do not see it as a normal thing. You know with some people; it is the life their child wants…. There are some children who do not pay heed even after several warnings, and there’s nothing anybody can do but to leave them alone. But it’s not something the community accepts or is happy about.* (AK Community Leader, SW, urban)

This community leader in the rural North West region specifically states that premarital adolescent pregnancy was something that brought shame to the girl and to her family. In this case, while there was a preference for early marriage and childbearing, unmarried girls were not expected to engage in sexual activity.

*It is a very shameful thing here for one’s daughter to get pregnant without marriage…. although it is very rare in our community and God forbid, we don’t wish it.* (MJ Community Leader, NW, rural)

This older mother expressed the opinion that adolescent pregnancy was desirable, as it enabled girls to have children early. She then stated a caveat, that it was only accepted when the girl was married.

*There is nothing wrong about it, even our parents some of them they married early and they gained so many things from there, you will see them and see their children you will not even see the difference, they will be young and their children will grow up together, when you see them in future, they will be able to gain so many things before they age… For unmarried girls it is not good, the person should finish school before marriage…but if she is her husband’s house, then better, it is good, there is nothing wrong in that one.* (OW Mother 01, SE, urban)

#### Support during pregnancy

Adolescent mothers in this study were largely dependent on their families for economic and emotional support to enable them cope during their pregnancies. The study showed that unmarried adolescent mothers relied mainly on their mothers or older female relatives for such assistance. The responses show in general the importance of parental, familial and spousal support for adolescent mothers, as they tend to be disadvantaged financially, emotionally and socially due to their young age.

This adolescent mother, while living with her family, was able to get quite a lot of moral and financial support from her brothers, and her older female relatives as both her parents were dead. In addition, she also got such support from her baby’s father.

*I got money from my older brothers as well as my baby’s father…. My family treated me well, they did not maltreat me because of the pregnancy…...My aunties were there for me, they gave me advice and moral support as well as money.* (AS02, 19, single, SE, Igbo, rural, Christian, incomplete basic)

While this adolescent mother was able to get support from her parents, she got nothing from her ex-husband and his family. This shows the importance of parental support especially in the case of marital breakdown or where the child’s father refuses to take responsibility for the mother and child.

*My parents helped me during and after pregnancy. My husband and his people did not care at all.* (KT05, 19, divorced, NW, Hausa, urban, Muslim, incomplete secondary).

These two adolescents have similar stories of abandonment by their babies’ fathers. While the first participant said that they had no plans to marry each other, the second participant said that her partner refused to support her because her parents refused to allow him marry her. These responses give insight into how relationship dynamics among adolescent mothers and their partners may influence the availability of partner support for young unmarried mothers.

*My baby’s father does not give me money. It’s my mother that takes care of me because we did not plan to marry each other.* (AK04, 19, single, SW, Yoruba, urban, Christian, incomplete secondary)

*He has abandoned me, saying that it was my parents that didn’t agree to accept him, and since then he has not been calling or taking care of me or the baby.* (AP04, 21, single, SW, Yoruba, rural, Christian, secondary)

This adolescent mother reported that she was abandoned by both her partner and her mother, so she was forced to fend for herself throughout her pregnancy. As earlier stated, some parents abandon their daughters when they get pregnant in a bid to force them into premature adulthood, as they believe that the girl’s getting pregnant is a statement that she is now an adult, and so should be treated as such.

*My mother did not treat me well during my pregnancy…she kept me at arms’ length.* (AK06, 19, cohabiting, SW, Yoruba, urban, Christian, basic)

On the other hand, married and cohabiting mothers relied more on their partners and partners’ families for all types of support.

*It was my husband that was taking care of me…He abandons his work to take care of me sometimes.* (KT08, 18, married, NW, Hausa, urban, Muslim, incomplete secondary)

*During my pregnancy my husband took care of me, provided my needs very well together with his parents.* (MJ05, 15, married, NW, Hausa, rural, Muslim, none)

*My husband and my mother-in-law are the ones who give me money for my needs, including hospital.* (OW01, 18, married, SE, Igbo, urban, Christian, secondary).

These adolescent mothers reported that they received adequate support from their partners during their pregnancy, as their husbands catered to their every need. Two adolescent mothers reported that they received support not only from their partners but from their in-laws also.

#### Experiences with health workers

There were some accounts of ill-treatment by health workers, ranging from being duped by the health worker to verbal abuse and carelessly causing the death of a respondent’s newborn baby.

This adolescent mother reports that she got scammed by a health worker in the government clinic, who collected money from her and promised to attend to her maternal health needs, However, she failed to do so and simply disappeared with the money paid, forcing the young mother to find an alternative healthcare service provider. While we cannot infer that this was a regular practice in the government health centre, it is doubtful that the said health worker would have pulled it off with a more matured and experienced client.

*The first clinic I went to…the lady took my money and disappeared…she never treated me. That was the government clinic…every time I went there, she was not around and whenever I tried to phone her, I wouldn’t get through. I eventually gave birth to my baby in a private clinic.* (AS03, 17, formerly cohabiting, SE, Igbo, rural, Christian, incomplete secondary)

This respondent complained about verbal abuse from the nurses at her antenatal clinic.

*They are quite expressive and sharp-tongued. They insult us a lot. If we don’t do our exercises on time they ask if they are the ones responsible for our pregnancy. They insult us like that.* (AK07, 20, cohabiting, SW, Yoruba, urban, Christian, secondary)

This respondent’s complaint was a serious one, as she claimed that the health worker at the government clinic was responsible for the loss of her first baby shortly after delivery.

*I don’t like the way they behave in that government clinic; they are careless people. I lost my first baby there because the nurse was careless.* (AS12, 19, cohabiting, SE, Igbo, rural, Christian, basic)

A common thread through these narratives is the lack of respect for these mothers by the health workers. However, despite these reported cases of negative health worker attitudes, the adolescent mothers who made the complaints still reported that they made use of health facilities for their maternal healthcare needs. This finding shows that as much as positive health worker attitudes make accessing healthcare less stressful for adolescent mothers who make use of health facilities, negative attitudes do not influence their decision to use health facilities. Rather, these decisions are influenced by other factors.

### Maternal healthcare utilisation by adolescent girls

Adolescent mothers reported different factors that influenced their use and non-use of maternal healthcare services for prenatal and delivery care.

#### Affordability and misinformation about cost of care

Some of the adolescent mothers reported that they were unable to make use of modern healthcare services because they could not afford the cost of healthcare, because they had no financial support, or they were misinformed about the true cost of healthcare for pregnant women.

*You know, those that use hospitals are those that can afford them, to know their condition…I was told that the hospital was expensive then, so I chose to use the ‘mission’…I couldn’t afford the cost of the hospital that was why I decided to go to the ‘mission’.* (AK 02, 16, single, SW, Yoruba, urban, Christian, incomplete secondary).

The “mission house” mentioned by this respondent refers to unlicensed maternity centres which are run by some churches. These centres are often staffed by women who have little or no formal training in pregnancy and delivery care who often learn their craft via experience. These alternative healthcare practitioners are a common feature in the urban South West zone. Some young mothers erroneously assumed that the “mission houses” were cheaper than modern health facilities but this was not always the case, as evidenced by one of the mothers whose daughter made use of the health centre for her maternal healthcare needs.

*The money they collect is not much. When my child gave birth, they collected #2,000* (about US$4) *from me because the things we were to bring were not complete. Their fee normally is #1,000* (about US$2) *but we didn’t take sanitary pads along, that’s why we paid #2,000. So, their money is not that expensive.* (AP Mother 01, SW, rural)

The responses below show the situation of some adolescent girls, particularly the unmarried ones, who were unable to make use of any form of healthcare during their pregnancies because they lacked a means of financial support.

*I didn’t go for any health care because my mother is not financially buoyant so I did not register in any hospital, mission or do any scan.* (AK04, 19, single, SW, Yoruba, urban, Christian, incomplete secondary)

*I didn’t have money for clinic because my mother kicked me out… I am grateful that I have my baby here. I thank God…. No, I didn’t attend the clinic at all throughout my pregnancy.* (AK05, 19, single, SW, Yoruba, urban, Christian, incomplete basic)

*I didn’t attend the clinic because there was no money for me to pay* (AS10, 18, single, SE, Igbo, rural, Christian, basic)

*I am not going (sic) anywhere, because that time no help, nobody is helping me (sic).* (AS09, 19, widowed, SE, Igbo, rural, Christian, incomplete secondary)

#### Healthcare preferences

Individual healthcare preferences were found to influence maternal healthcare usage, and were observed to be shaped by religious beliefs, community and family preferences. Some adolescent mothers preferred using traditional healers and “mission houses”, others preferred using modern health facilities, and yet others used a mixture of the two.

*There was a day I visited a friend who just gave birth and the midwife who took her delivery saw a vision about my friend and I, that we should do a special bath for spiritual cleansing. So, when I was in labour I told my people to take me to the woman at the mission. So, it was the woman who was helped me on that day when I delivered my baby.* (AK04, 19, single, SW, Yoruba, urban, Christian, incomplete secondary)

The above response shows the importance of religious influences on the type of healthcare patronised by pregnant adolescent girls. This young mother reported that she delivered her baby at a “mission house” rather than a health facility due to a prior “prophecy” given to her during her pregnancy.

Some other adolescent girls reported that they made use of both traditional and modern healthcare service providers during their pregnancies. A key to their choice to make use of both types of healthcare is found in the participants who said that they had either heard of the efficacy of herbal methods, or were advised to make use of certain healthcare providers by persons they had relationship with in the community.

*I used both modern and traditional healthcare…because I like the two…I was going to the health centre and using herbs also…they made me and the baby to be strong.* (AS02, 19, single, SE, Igbo, rural, Christian, incomplete basic)

*Yes, when I was pregnant, I took herbs…they said it aids easy delivery and widening of the (cervix) during labour.* (KT03, 18, married, NW, Fulani, urban, Muslim, no education).

*I gave birth at home. A woman in the community assisted me during the child birth. After I gave birth, she advised that we go to the hospital for proper check up to ascertain my health and that of the baby. We were all ok and drugs were given for me and the baby.* (MJ05, 15, married, NW, Hausa, rural, Muslim, none)

These responses also show that their choices of maternal healthcare provider were influenced by what was perceived to be the norm in their environment.

It is evident from the narratives is that mothers are an important influence over their daughters’ choice of healthcare provider, as the daughters tended to use the same type of provider as their own mothers did, and possibly suggested to them to use. The daughters of the two women interviewed below made use of the same type of healthcare providers that their mothers used in their own childbearing period.

*When I was still giving birth; I didn’t go to the hospital. I went to the church till I gave birth to my four children. What took me to the hospital is the immunisation that I took them to the hospital for.* (AK Mother 01, SW, urban)

*That time was better than now… Although many of my children I delivered them in private hospital …Yes, my daughter also delivered in a private hospital.* (AS Mother 01, SE, rural)

## Discussion

The study examined the pregnancy experiences of adolescent mothers in Nigeria, as well as their maternal health-seeking behaviour, and the influences from the sociocultural environment that guided their choices. Understanding the circumstances that influence adolescent mothers’ maternal healthcare would go a long way to help design interventions to increase usage of modern health services among these young mothers in Nigeria. This will enable appropriate action to be taken in reviewing the present policies on adolescent health and maternal health service provision in Nigeria.

The study found that a large number of unmarried adolescent girls reported that their pregnancies were unplanned, while married girls majorly reported having planned and expected pregnancies [10, 11]. Among unmarried girls, this finding is a cause for concern, as it gives evidence that they are engaging in sexual intercourse without the required knowledge, motivation and means to prevent unwanted and mistimed pregnancies [12, 22]. The study also revealed that premarital adolescent pregnancy was stigmatised, as many respondents reported being verbally abused or harassed by their community members or even complete strangers, just as previous African and global studies have found [11, 29, 31, 32, 42, 58]. Additionally, most married and cohabiting adolescent girls regardless of their age reported no stigma, showing that being in a recognised union had a protective effect against stigma for adolescent mothers in Nigeria.

A good number of adolescent mothers who experienced stigma still made use of modern health facilities for their maternal health needs, showing that it did not necessarily hinder their using health facilities, in contrast to previous studies by Agunbiade et al. and Chikalipo et al. [11, 33]. Inasmuch as stigma did not influence maternal health care use by some of the adolescent mothers, it may have caused them psychological stress, which may affect their health and the health of their unborn babies.

The study discovered adolescent girls relied mainly on their relatives for social and financial support. For unmarried adolescents, they often relied on their mothers to provide these kinds of support, and where their mothers were either unable or unwilling to provide this support, some relied on another older female relative for support. Married adolescent mothers, on the other hand, tended to receive support from their partners and in-laws [34, 38]. The findings suggest that married adolescent mothers had a wider network of support than their single counterparts. Adolescent mothers were, due to their lack of experience, largely dependent on their families and /or partners for emotional support. Adolescent mothers who reported an ample amount of support from their families, especially their mothers and partners, were able to make use of maternal healthcare, unlike those who reported being abandoned during their pregnancies. This finding goes to show the importance of family support in ensuring that adolescent mothers are able to access and use adequate maternal healthcare. Abandonment by partners was a common experience reported by the single adolescent mothers. This is likely because their partners were adolescents and young adults with limited financial capacity themselves, and were unable to take care of them and their babies.

Similar to other studies [28, 31, 32, 34–37], some adolescent mothers reported that health workers had negative attitudes towards them. It was discovered that adolescent girls who experienced bad treatment at the hands of health workers were noticeably reluctant to continue patronising health facilities. These findings imply that health workers may need to be trained to provide respectful and non-judgemental care to adolescent mothers.

The study participants reported different maternal healthcare service usage patterns. While some of them made use of modern health facilities, others made use of traditional healthcare practitioners, and yet others combined both modern and traditional maternal healthcare, while some girls were unable to make use of any form of maternal healthcare at all. For those who were unable to make use of modern healthcare services, some reported a lack of financial support to pay for medical bills, while some girls reported that they believed that they would be unable to afford the cost of healthcare due to misinformation. This shows that financial support was an enabler for modern healthcare access for pregnant adolescent girls, as girls who were unable, or believed they were unable to afford the cost of modern healthcare did not use it, rather relying on alternative sources of healthcare. Affordability is a major constraint to the use of maternal healthcare in Nigeria, as in many low- and middle-income countries, and the problem of affordability is worst for adolescent girls who have no livelihood, are not in a union, lack social support, and are from low-income families [20–24, 34, 38].

Maternal and community healthcare preference influenced the type of providers adolescent mothers used, and they conformed to these decision makers’ preferred healthcare providers [28, 39]. Mothers were discovered to influence their daughters’ maternal healthcare choices, and they tended to support and enable their daughters’ choices. Cultural influences also determined the types of maternal healthcare that were favoured by adolescent mothers, as adolescent girls tended to follow the type of healthcare practiced in their communities, whether it was modern, traditional or complementary. Religious leaders are important healthcare decision makers for some adolescent mothers, as some mothers reported that they were advised by these leaders to use “mission” houses, rather than health facilities for their maternal healthcare needs.

## Practical and theoretical implications of the study

The study found that in addition to social support, stigma, health worker attitudes and healthcare preferences as influences for adolescent maternal healthcare utilisation, the role of maternal social support (both emotional and financial) and the influence of religious leaders were found to be important. Also, finances were found to play a major role in whether or not adolescent mothers made use of maternal healthcare services. Misinformation concerning the cost of healthcare services was found to serve as a barrier to the usage of such services for some pregnant adolescents.

The study found that individual and community healthcare preferences were predisposing factors for maternal healthcare use among adolescent mothers. The presence of social support was an enabling factor for healthcare service use, while the perception of need for maternal healthcare services among adolescent mothers, their own mothers and the wider community was discovered to be the important need factor for use of maternal healthcare among adolescent mothers.

## Limitations of the study and areas for future research

Due to the purposive sampling procedure used to select participants, it is recognised that the sample may not be representative of the entire population of adolescent mothers in Nigeria. Also, findings from each location and the study as a whole may not be generalisable to the entire population of the country. As such, future research can examine the sociocultural influences that guide adolescent maternal healthcare use in other contexts within and outside Nigeria. Future research can also expand the study sample size and utilise the findings of this study to create testable hypotheses (Additional file 1).

## Conclusion and recommendations

This paper brings attention to the experiences of adolescent mothers in Nigeria, as well as their maternal healthcare usage patterns and the sociocultural factors influencing their use of these services. The study discovered that sociocultural factors such as presence of social support and the type of family and community healthcare preference influenced maternal healthcare utilisation among adolescent mothers, while pregnancy intention, stigma and negative health worker attitudes had little influence on maternal health-seeking behaviour. The prevalence of unwanted pregnancies among adolescent mothers suggests the need for reproductive health programmes that enable sexually active adolescent girls to delay and prevent pregnancies. The role of social support was an important one, as adolescent mothers who had support from their close family members were able to make optimal use of healthcare services, compared with those who had little or no support. Therefore, the role of maternal and partner support must be emphasised in programmes concerned with increasing maternal healthcare utilisation among adolescent mothers. As community and religious healthcare preferences influenced choice of healthcare provider for young mothers, healthcare interventions must strive to be culturally sensitive so as to be acceptable to young mothers. Religious and cultural leaders also need to be educated on the benefits of adolescent mothers receiving maternal healthcare from recognised health facilities, and be enrolled in the effort to ensure that adolescent mothers make use of these services. Study findings underscore the need for adolescent-friendly maternal health services and training programmes that ensure that health workers provide respectful and non-judgemental care to adolescent mothers. As stigma against adolescent mothers was still very present in the study areas, behavioural change education needs to be targeted towards the wider public to eradicate stigma towards younger mothers.

## Supplementary Information

**Additional file 1**: Interview protocol.

## Data Availability

Data supporting the findings of the study are contained within the manuscript.

## Abbreviations

AK: Akure
AP: Aponmu
AS: Assa
KT: Katsina
MJ: Majigiri
OW: Owerri
NW: North West
SE: South East
