# Supplementary material for: A qualitative investigation into pregnancy experiences and maternal healthcare utilisation among adolescent mothers in Nigeria
Source: Reprod Health. 2023 May 20;20:77. doi: 10.1186/s12978-023-01613-z (PMC10199580; doi:10.1186/s12978-023-01613-z)
Supplement: Supplementary file 1 — Additional file 1: Interview protocol. [file 12978_2023_1613_MOESM1_ESM.docx]

**Interview Protocol**

| **S/N** | **Questions** |
| --- | --- |
|  | Were you planning to get pregnant when you did? If not, can you tell me what happened? |
|  | How is adolescent pregnancy viewed in this community? Is it accepted or seen as shameful? |
|  | Did you get any form of support during the pregnancy from your family and/or partner’s family (Probe for: financial support, emotional support, advice, help, transport and so on)? |
|  | How do the health workers treat you? Have you ever had bad treatment from them? |
|  | What type of healthcare have you used/did you use during your pregnancy? Why did you use this particular type of healthcare? (Probe for: access, cost, convenience) |
|  | What have been your own experiences with maternal healthcare use in the past? (Probe for: cost, distance, quality of care services, health workers) |
|  | Were you planning to get pregnant when you did? If not, can you tell me what happened? |
|  | What type of healthcare do you prefer during pregnancy? Probe for: pluralism in care providers (use of both orthodox and traditional care practices). |
|  | What type of healthcare have you used/did you use during your pregnancy? |
|  | Why did you use this particular type of healthcare? Probe for: access, cost, convenience. |
|  | Did you use antenatal care? Where did you do it? How many times did you go? Can you tell me some of the services that were given to you? |
|  | How do the health workers treat you? Have you ever had bad treatment from them? Probe for: rudeness/disrespect/name calling, bad service. |
|  | Where did you give birth? Who assisted you during delivery? Why did you choose this particular person/place? What was your delivery experience like? |
|  | Where do you intend to give birth? Why did you choose this option? Probe: accessibility, cost, convenience. Did you choose it on your own or did someone make the decision for you? |
|  | After giving birth, where did you and the baby go for post-delivery checkup? Who attended to you? Did you choose this person yourself? Who made the decision for you? |
|  | What type of healthcare is preferred in this community? Probe for: traditional, orthodox, pluralistic healthcare use. |
|  | Do you think that pregnant adolescents require healthcare? Why? What type of healthcare would you suggest that pregnant adolescents use? Why? |
